# Respiratory syncytial virus (RSV): over 60 years of research but still so many unanswered questions

**DOI:** 10.1177/20499361231159991

**Published:** 2023-03-21

**Authors:** Simon B. Drysdale, Lindsay Broadbent

**Affiliations:** Department of Paediatrics, St George’s University Hospitals NHS Foundation Trust, London, UK; Centre for Neonatal and Paediatric Infection, St George’s, University of London, London, UK; School of Biosciences and Medicine, University of Surrey, Guildford GU2 7XH, UK

Respiratory syncytial virus (RSV) infection remains an unmet medical need. It is responsible for an estimated 33 million acute lower respiratory tract infections (LRTIs) and over 3 million hospitalisations in children below 5 years old worldwide each year.^1^ In addition, older adults and immunosuppressed patients are also at high risk of severe disease. Despite decades of research, our understanding of RSV virology and pathogenesis is still incomplete. Recent years have brought significant advances in the development of potential vaccine candidates, but there are currently no licensed vaccines or effective treatments, though monoclonal antibodies are available to protect high-risk infants.^2,3^

This special edition of *Therapeutic Advances in Infectious Disease* pulls together experts in the field to broadly explore important aspects of RSV research with potential clinical application.

RSV is a single-stranded, negative-sense RNA virus of the genus *Orthopneumovirus*, family *Pneumoviridae.* Transmission is primarily through exhaled microdroplets. RSV infects the ciliated epithelium of the respiratory tract, resulting in inflammation, leukocyte recruitment, increased mucus production and airway epithelial cell sloughing.^4,5^ Severity of disease is likely due to a combination of both viral and host factors.^6^ Several putative receptors for RSV have been investigated including CX3CR1, nucleolin and epidermal growth factor receptor (EGFR).^7,8^ Further work is needed to understand if identified receptors or host factors driving viral replication could be a focus of future pharmaceutical development. In this issue, Bergeron *et al.*^9^ showed that in a mouse model, treatment with an anti-G protein mAb (3D3) that blocks G protein CX3 C-CX3CR1 interaction resulted in improved interferon responses compared with palivizumab following RSV infection. Most therapeutics currently in development target the RSV fusion (F) protein and this study suggests a novel pathway for therapeutic development.

RSV is a major pathogen of young infants accounting for up to 125,000 deaths worldwide each year, particularly in low-income countries.^10^ In addition to acute respiratory disease, moderate-severe RSV infection in early life is associated with the development of wheeze and asthma. The mechanisms underlying the correlation between RSV infection and asthma are still under investigation. Difficulty in diagnosing asthma often results in under diagnosis, particularly in low- or middle-income countries (LMIC), as highlighted in this issue by Islam *et al*.^11^ While many prospective studies on RSV hospitalisation have been published, it is important that this work continues. Identifying changes in disease incidence, severity and temporality can influence protocols for clinical management and, ultimately, improve patient care. Wright *et al.*^12^ found that infants born prematurely present to healthcare providers with RSV earlier compared with term born infants (47% *versus* 37% cases within the first 3 months). In addition, preterm infants accounted for 20% of RSV-related hospitalisations, but only accounted for 8% of births within the same region. The reasons for this are not fully established but could be due, in part, to immature immune responses. Future prospective studies are likely to assess the impact of the COVID-19 pandemic on many aspects of RSV epidemiology and pathogenesis. Understanding infection dynamics of RSV is crucial to the development of new clinical strategies to prevent severe disease.

In addition to causing significant disease in young children, RSV is increasingly recognised for its impact on older adults and individuals with compromised immunity, such as transplant recipients. In these individuals, RSV can quickly progress to severe LRTI resulting in pneumonia, loss of a transplanted organ, or even death. There are currently no specific treatments for RSV infection for most patients with RSV infection and care is usually supportive with oxygen and fluid support. Ribavirin, a nucleoside inhibitor, has been available for many years and has activity against RSV, though evidence for its efficacy is limited and it has a poor side effect profile.^13,14^ As Villanueva *et al.*^15^ highlight in their article in the series, there is some evidence for its use in reducing morbidity and mortality, along with intravenous immunoglobulin, in specific high-risk populations such as adults who are immunocompromised having undergone organ or hematopoietic stem cell transplant. However, new antivirals are needed, and there are several in the pipeline undergoing phase II and III clinical trials.

Palivizumab, an RSV-specific monoclonal antibody, has been available as preventive therapy for many years, but due to its high cost and need for monthly injections, it is only used in very-high-risk infants. Importantly, Rankin *et al.*^16^ found that 88% of infants presenting to a variety of healthcare settings with RSV illness were not preterm and 92% had no preexisting conditions. Novel RSV monoclonals to prevent severe RSV infection in infants are in late-stage clinical trials, with one (nirsevimab) recently approved by the European Medicines Agency (EMA) and UK Medicines and Healthcare products Regulatory Agency (MHRA). In addition, multiple RSV vaccines are in late-stage development with target groups including infants, pregnant women and older adults.

The COVID-19 pandemic has increased the body of evidence around respiratory virus transmission dynamics, nonpharmaceutical interventions and outbreak monitoring. As discussed by Guzman and Hultquist,^17^ our understanding of RSV genetic diversity is incomplete. Investment in molecular surveillance of RSV genotypes and viral evolution could be correlated with seasonality and disease severity. With the advent of new vaccines and therapeutic strategies, monitoring of viral evolution will be of upmost importance to identify and understand any evolutionary pressure on RSV.

## Conclusion

RSV infection continues to cause a significant burden on healthcare resources. The scientific community has witnessed unprecedented collaboration and focus to tackle the SARS-CoV-2 pandemic. New interest, enthusiasm and lessons learned as well as novel protocols, technologies and collaborations that were essential for the rapid pace of knowledge exchange during the pandemic will hopefully translate to other areas of infectious disease research such as RSV. The ability to then pull together all aspects of RSV research, from basic science to epidemiology and drug development, will all contribute to the understanding of RSV disease and the impact of clinical care as shown in the range of articles in this Special Edition. In view of the ongoing work is possible, we will see a huge surge in RSV research, vaccine candidate and therapeutic treatment development in the near future with the resultant improvement in patient care.

## Declarations

**Ethics approval and consent to participate:** Not applicable.

**Consent for publication:** Not applicable.

**Availability of data and materials:** Not applicable.
